# Management of complex orthodontic cases using aligners: A comparative study

**DOI:** 10.6026/973206300221429

**Published:** 2026-03-31

**Authors:** Mohammed Ali Hamid Syed, Chaitanya Chappidi, Zeeshan Mubashir, Vijay Shekhar, Lahari Priya Singareddy, Rahul Tiwari, Heena Dixit Tiwari

**Affiliations:** 1 Department Orthodontics and Dentofacial Orthopedics, Al harkan Medical Center, Unaizah, Al Qassim, Saudi Arabia; 2 Department of Dental Medicine, Metro Health Family Dentistry, Cleveland, Ohio, United States of America; 3 Department of Orthodontics and Dentofacial Orthopaedics, Al Jouf Specialised Dental Center, Ministry of Health, Sakaka, Al Jouf Province, Kingdom of Saudi Arabia; 4 Department of Dentistry, Nalanda Medical College Hospital, Agamkuan, Patna, Bihar, India; 5 Department of Public Health, St. Francis College, New York, United States of America; 6 Department of Dental Research Cell, Dr. D. Y. Patil Dental College & Hospital, Dr. D. Y. Patil Vidyapeeth (Deemed to be University), Pimpri, Pune 411018, Maharashtra, India; 7 Department of blood cell, Consultant, Blood Cell, Commissionerate of Health and Family Welfare, Government of Telangana, Hyderabad, Telangana, India

**Keywords:** Clear aligner, fixed appliance, complex malocclusion, peer assessment rating (PAR), Oral Health Impact Profile-14 (OHIP-14)

## Abstract

Clear aligners are increasingly being used for complex malocclusions; however, adequate outcome data comparing them with fixed appliances in high-difficulty cases is still limited. Therefore, it is of interest to compare occlusal outcomes, efficiency and patient-reported experience between clear aligners and fixed appliances in complex orthodontic cases. Using a prospective comparative design, complex cases were randomly assigned to treatment with clear aligner therapy or conventional fixed appliances and assessed by Peer Assessment Rating (PAR) score change, length of treatment time needed, burden of appointments required, refinements/finishes and OHIP-14. Both modalities produced clinically significant occlusal correction; however, fixed appliances showed increased efficiency in extraction heavy biomechanics while aligners showed increased initial patient-reported comfort and oral health related quality of life. Aligners may be useful in complex cases when biomechanics has been well-staged and compliance is high and yet fixed appliances might still be beneficial in providing efficiency benefits in hard-to-root control and extraction space closure.

## Background:

Complex orthodontics cases are usually the ones that have severe crowding, extraction mechanics, transverse and vertical discrepancies and a high demand of root control. These features form a clinical stress test to the effectiveness of any appliance as well as their predictability. Certain more recent randomised data have actually shown that aligners are able to relieve the load of OHR-QoL at the outset of the treatment versus conventional functional appliances-even in severely crowded cases [1]. These appliances need to be, however, perceived in connection with biomechanical constraints and finishing requirements. There are comparative data studies on mixed and adult Class II malocclusiveness that show that aligners applied to the maxillary arch is capable of achieving the same sagittal correction as fixed appliances. This is specific in selected scenarios, with potentially better control of lower incisor proclination [2, 3]. But, predictable expression of planned movements (especially rotation/torque) is variable. The treatment planning often requires over treatment, auxiliaries and refinement cycles [4, 5]. Therefore, it is of interest to compare aligners versus fixed appliances and specifically in complex case management using standardized occlusal and patient-reported outcomes.

## Materials and Methods:

This prospective comparative research was performed in a university-affiliated orthodontic clinic. The patients aged 16-35 years coming with complex malocclusion were enrolled. The complexity defined as the presence of any two of the following criteria: severe crowding (≥6 mm) in at least one arch, a premolar extraction treatment plan, clinically significant rotation/torque demands (≥15°C rotation or planned incisor torque correction ≥8°C), deep bite or open bite requiring vertical control mechanics, or clinically relevant asymmetry requiring staged mechanics. Participants were allocated into two equal groups (n=30 each). The aligner group received clear aligner therapy with staged movement planning where auxiliaries such as attachments and elastics were permitted and refinements were allowed. The fixed appliance group was treated using preadjusted edgewise fixed appliances with conventional mechanics, with elastics and adjunctive auxiliaries used as required. The primary outcome was change in Peer Assessment Rating (PAR) score from baseline to completion of active treatment. The secondary outcomes included total treatment duration (months), number of clinical visits, number of refinement/ finishing phases (aligners) or finishing wire phases (fixed appliances) and Oral Health Impact Profile-14 (OHIP-14) scores assessed at one month and at treatment completion. Between-group comparisons were performed using independent t-tests or Mann-Whitney U tests for continuous variables and chi-square tests for categorical variables, with statistical significance set at p<0.05.

## Results:

Baseline comparability was achieved between groups, with no statistically significant differences in age, sex distribution, baseline PAR score, or the complexity features used for case definition. Severe crowding was present in almost two-thirds of patients. In half required premolar extractions. This shows that a clinically demanding sample. The prevalence of rotation/torque-intensive mechanics was also balanced, supporting a fair comparison of efficiency and finishing requirements. Both cohorts represented high-difficulty orthodontic case management Table 1 (see PDF). Both the groups achieved substantial occlusal improvement, with high PAR reduction and low final PAR scores and the between-group difference in PAR change was not statistically significant. Fixed appliances had significantly shorter treatment duration. This was in accordance with the effectiveness of sustaining the complicated mechanism. This was in accordance with the effectiveness of s ustaining the complicated mechanism. The aligners had more stages of refinement. The number of overall clinic visits required with aligners was lower though. This is a protocol based tray progression that includes targeted re-planning of them. The patient-reported impact at 1 month (OHIP-14) was also significantly less in the aligners. The two modalities did not show any significant differences Table 2 (see PDF).

## Discussion:

The given study proposes that clear aligners could offer clinically significant occlusal change in complicated orthodontic situations. Nevertheless, the efficiency and finishing behaviour was proved to be different than that of fixed appliances. The increased treatment time with aligners is also likely due to the refining stages that are necessitated by the movement of teeth not reaching the virtual plan well, especially when it involves the making of difficult movements like rotations and torque. Attachment design and location are also very significant in biomechanical rotation control. The finite element investigations have demonstrated that properly mounted attachments, particularly paired lingual and buccal attachments, enhance aligner retention thereby resulting in an efficient tooth movement compared to none attachments [6]. This finding can verify our observation of more refinement steps in the aligner group in which clinicians tend to add to the laggard expression the staged re-planning. Aligners were initially more preferred by patients, demonstrated by the reduced OHIP-14 scores at one month. This is equivalent to the evidence of trials that aligner thickness, material and the method of forces delivery may affect the pain levels and patient satisfaction in the long term [7]. Nevertheless, in more complicated situations, effective therapy usually requires good root management, solid torque manifestation and bodily tooth motion under control during closing of the space- where aligners are still less depictable. In this group, patients that had to have significant adjustments on the torque and rotation finalized plenty of refining phases. Clinical reports supporting this include that it has been observed that the planned tore changes can be insufficient to express themselves and can also imply undesirable tipping when staging and auxiliary mechanics are not carefully planned [8]. Tooth derogations are also less predictable and most times requires scheduled overtreatment to achieve the desired final position. The precision is based on the kind of tooth and the extent of the rotation and careful planning and well-designed finishing protocol is also significant in maintaining consistent outcomes [9]. Safety and periodontal outcomes matter in complex extraction cases with significant incisor retraction. Recent CBCT-based comparative studies document differences in root resorption patterns and changes to the incisive canal between aligners and fixed appliances; therefore, biologic monitoring and limits on tooth movement are crucial in high-demand mechanics [10, 11-12]. Other evidence indicates measurable changes to alveolar bone as well as differences in root resorption under extraction and non-extraction protocols for appliance systems, supporting individualized risk counseling within complex orthodontic treatment plans [10, 11-12]. Expert consensus places importance on case selection, difficulty grading, and standardized management strategies for off-tracking (such as elastics and segmental mechanics) to improve outcomes in higher-difficulty aligner cases [13, 14]. These expert recommendations also provide structured clinical decision support for aligner use when biomechanics must be coordinated with patient compliance and anchorage strategies [13, 14]. Finally, recent evidence synthesis indicates that although both appliance systems can achieve treatment objectives, fixed appliances may retain an advantage in extraction-based complexity and scenarios requiring precise control of tooth movement [15]. Recent clinical and imaging-based investigations have further examined biomechanical predictability, periodontal responses and root resorption patterns during orthodontic treatment with aligners and fixed appliances, emphasizing the importance of careful treatment planning and biologic monitoring in complex cases [10,11- 12]. Furthermore, contemporary systematic evaluations and expert consensus studies highlight that successful aligner therapy in high-difficulty malocclusions depends on appropriate case selection, auxiliary mechanics and patient compliance to achieve outcomes comparable to conventional fixed appliance therapy [13, 14-15].

## Conclusion:

Clear aligners and fixed appliances are equally capable of delivering an acceptable level of occlusal enhancement in complicated orthodontic patients. Fixed appliances provide similar correction, though generally in a much reduced total treatment time. Nevertheless, aligners are more likely to have a higher short-term effect and less adverse effect on OHIP-14 at the beginning of treatment. With problematic biomechanics, aligner success requires meticulous staging, properly designed auxiliaries and refinements planning to achieve the stage of efficiency that can be more reliably provided by fixed appliances.
